# Health and Well-Being in the Context of Health-Promoting University Initiatives: Protocol for a Mixed Methods Needs Assessment Study at Qatar University

**DOI:** 10.2196/58860

**Published:** 2024-12-18

**Authors:** Ghadir Fakhri Al-Jayyousi, Diana Alsayed Hassan, Hanan Abdul Rahim, Manar E Abdel-Rahman, Isabel Ferreira, Banan Mukhalalati, Lily O'Hara, Hanan Khalil, Reema Tayyem, Elham S Abu Alhaija, Randa Abidia, Monica Zolezzi, Alla El-Awaisi, Noor Al-Wattary, Rafif Mahmood Al Saady, Maguy Saffouh El Hajj, Mujahed Shraim, Arpi K Abouhanian, Hatoun Saeb, Mustapha Mohammed, QU Health Youth Wellbeing Research Network Group

**Affiliations:** 1 Department of Public Health, College of Health Sciences, QU Health Qatar University Doha Qatar; 2 Department of Rehabilitation Sciences, College of Health Sciences, QU Health Qatar University Doha Qatar; 3 Vice President for Medical and Health Sciences Office, QU Health Qatar University Doha Qatar; 4 Department of Nutrition Sciences, College of Health Sciences, QU Health Qatar University Doha Qatar; 5 College of Dental Medicine, QU Health Qatar University Doha Qatar; 6 Department of Public Health Dentistry, College of Dental Medicine, QU Health Qatar University Doha Qatar; 7 Department of Clinical Pharmacy and Practice, College of Pharmacy, QU Health Qatar University Doha Qatar; 8 College of Education and Arts Lusail University Doha Qatar; 9 Department of Basic Sciences, College of Medicine, QU Health Qatar University Doha Qatar; 10 Office of Chief Strategy and Development Qatar University Doha Qatar; 11 Health in all Policies Unit, Minister of Public Health Office Ministry of Public Health Doha Qatar; 12 QU Health Qatar University Doha Qatar

**Keywords:** college students, needs assessment, qualitative, lifestyle behaviors, environmental scan

## Abstract

**Background:**

Health-promoting universities are dedicated to fostering learning environments and organizational cultures that support the physical and mental well-being of students, faculty, and staff. As students constitute the largest group within the university community, any policy intervention targeting them is likely to have a significant impact on the university as a whole.

**Objective:**

This study aims to assess the health status and needs of Qatar University (QU) students using a comprehensive and holistic definition of health, informed by the perspectives of students, faculty members, and key informants. The ultimate goal is to inform evidence-based policies and services designed to improve students’ physical and mental well-being.

**Methods:**

An explanatory sequential mixed-methods research design will be used to conduct a comprehensive assessment of students’ health status and needs. This assessment will consist of a quantitative component (a web-based health survey) administered to a convenience sample of students, and a qualitative component, including focus groups with students and faculty members, as well as interviews with key informants. Priority health issues and their determinants, identified through the quantitative assessment, will inform and guide the qualitative assessment to provide a deeper understanding of the various contexts and factors shaping them. Descriptive analyses (eg, proportions or means with SDs), comparative analyses (eg, t tests or chi-square tests), and association analyses (eg, linear, logistic, or Poisson regression models) will be used to analyze the quantitative data. Thematic analysis will be used in the qualitative assessments. Additionally, an environmental scan will be conducted to assess relevant facilities, services, and programs at the QU campus and the QU Primary Healthcare Corporation Center, as well as to review university policies and regulations that may affect students’ health and well-being. Together, the needs assessment and environmental scan will inform the design of multilevel interventions, including health education and promotion programs, health services orientation, and proposed policy changes.

**Results:**

Between March and December 2022, 812 students completed the web-based health survey. Data have been extracted, cleaned, and harmonized. Analyses to assess the extent of selection bias and the calculation of weights to account for this in all subsequent analyses have been completed (by December 2023). Following the completion of all quantitative data analyses (expected by the end of 2024), focus groups, interviews, and the environmental scan will begin in January-December 2025.

**Conclusions:**

This project will help identify and prioritize the health needs of QU students and their determinants, and inform relevant services and policies targeting these needs. By using comprehensive and context-appropriate methods, this project will contribute to QU’s strategic efforts to become a Health-Promoting University.

**International Registered Report Identifier (IRRID):**

DERR1-10.2196/58860

## Introduction

### University Students’ Health and Well-Being

University students are primarily individuals transitioning from adolescence to young adulthood. This is a critical developmental period marked by milestones and challenges that significantly influence overall growth, as well as lifelong health and well-being [1-4].

Several studies indicate that university students are less likely to engage in health-promoting behaviors, such as regular physical activity, healthy eating, proper sleep hygiene, stress management, and mental health care [2,3,5-10]. For example, Aceijas et al [10] reported that approximately half of the students at a university in London (United Kingdom) exhibited health-damaging behaviors, including insufficient physical activity, an unbalanced diet, and excessive alcohol consumption, alongside concerning levels of mental well-being. A large study investigating the health status of local and international students at an Australian university found that the vast majority did not meet health recommendations for physical activity and nutrition. Additionally, 26.5% of local students and 14.0% of international students reported having a mental health condition [11].

According to social-ecological theory, students’ health and well-being may be influenced by factors across multiple levels: intrapersonal (eg, age, gender, socioeconomic status, taste preferences), interpersonal (eg, peer pressure or support, lack of parental control), institutional (eg, campus built environment, schedules, policies), community (eg, student networks or events), and public policy (eg, state-regulated costs or support for tertiary education and housing) [12]. Specific challenges affecting students’ lifestyles—and consequently their physical and mental well-being—during the transition from secondary school to university include peer pressure or support, changes in school facilities [2-4], the quality of food courts [9], use and access to sports facilities [13], adjustment to new schedules and academic demands [2,3,8,14], development of new social networks [2-4], and financial instability (eg, tuition fees and student debt) [9,15]. These interpersonal, institutional-, community-, and policy-level factors highlight the critical role universities play in health promotion.

### Health-Promoting Universities

A Health-Promoting University is a system dedicated to enhancing and safeguarding the health and well-being of its community members through policies and practices that (1) foster social, cultural, economic, commercial, built, and natural environments that support and nurture the health, well-being, and sustainability of people and places; and (2) integrate health, well-being, and sustainability into the university’s curricula, teaching, and learning [16-18]. Since their inception in England in the mid-1990s, Health-Promoting University initiatives have been established in most regions of the world [19]. The Okanagan Charter, an international Charter for Health-Promoting Universities and Colleges, calls on these to “transform the health and sustainability of our current and future societies, strengthen communities and contribute to the well-being of people, places and the planet” [20]. Health-Promoting University initiatives are system-based approaches grounded in a socioecological framework. This framework emphasizes the importance of comprehensively addressing personal and interpersonal factors, as well as contextual and environmental factors (eg, policies or built environments) that shape the health and well-being of university community members [12]. These members include students, staff, faculty, and others affected by university operations. As students represent the largest single group hosted by universities, any health-promoting initiatives targeting students are likely to have a broader impact on the university as a whole.

Following best practices, a Health-Promoting University initiative should begin with an assessment of community needs and assets [21]. In a needs assessment study, students at Newcastle University in the United Kingdom expressed a desire for access to sports facilities, safe walking areas, and healthy food options on campus to support a healthy lifestyle [22]. Similarly, a study exploring the health-related needs of medical students in the United Arab Emirates identified several on-campus health literacy initiatives as essential for fostering a healthier student population [23]. Consistent with these findings, a study examining factors influencing health-related behaviors among university students in Jordan concluded that programs promoting healthy behaviors are necessary and should be tailored to the sociodemographic characteristics of the student body [24].

### Local Research and Gaps

In Qatar, most research conducted within the university context has focused on individual-level assessments of risk factors and their impact on health and well-being. Relevant to our study, these include studies examining the prevalence of overweight/obesity and unhealthy lifestyle habits [25], factors related to physical activity and sedentary behaviors [26,27], eating disorders [28], sleep quality and hygiene [29], tobacco use and cessation services [30-32], mental health [33-38], and health literacy (eg, inappropriate antibiotic use [39], screening programs [40], and COVID-19 vaccination [41]).

After reviewing the literature, we recognized the need to shift from an individual level to a more comprehensive assessment that includes the organizational, community, and policy levels. Such a holistic approach would provide the evidence needed to design and implement multilevel health promotion interventions, moving us closer to achieving a sustainable Healthy University. Aligned with this, a project was undertaken in 2019 to promote and update an existing policy prohibiting all forms of tobacco on campus, while also enhancing the availability of cessation support programs [42,43]. Additionally, contextual research was conducted to assess the educational needs and well-being of students during the COVID-19 pandemic [44-46].

In 2022, Qatar University (QU) was recognized as a Healthy University by the World Health Organization (WHO) following a thorough assessment of the Health-Promoting University indicators across its campus [16]. These indicators include, but are not limited to, initiatives promoting healthy lifestyles, mental health, reproductive health, communicable disease prevention, regular medical checkups and screening programs for students, the implementation and enforcement of national nutrition guidelines and the 100% tobacco-free policy, and the availability of qualified physical education professionals.

### Objectives

This study aims to assess students’ health and well-being, prioritize their needs and determinants, inform campus policies and services, and support QU’s efforts to maintain its status as a Healthy University. This will be achieved through the mobilization of the QU community, beginning with a comprehensive needs assessment that includes individual-, organizational-, and policy-level evaluations. The assessment will be followed by a response to the identified needs and gaps through appropriate multilevel interventions. The intervention activities will align with the pillars of Health-Promoting Universities: health development, equity, sustainability, and solidarity [16]. These pillars encourage universities to promote the health of their communities by integrating health concepts into their processes and policies.

- Objective 1: To conduct needs assessments from the perspectives of students and key informants to:
  Describe the health status and needs of QU students, specifically concerning physical and mental health, lifestyles (nutrition habits, physical activity, and smoking), oral health, health literacy, and health care utilization.
  Examine the association between students’ demographics (eg, gender, nationality, socioeconomic status) and various health status indicators and behaviors.
  Identify priority health issues to address based on burden, impact, and unmet needs.
  Assess the perceptions of the students, faculties, and key informants regarding the availability, utilization, and usefulness of various health services on the QU campus.
  Identify priority areas for interventions that are informed by students’ input and preferences, and are likely to have a meaningful impact.
- Objective 2: To conduct a campus environmental scan, including an assessment of the physical environment and an analysis of relevant policies.
- Objective 3: To plan and propose evidence-based intervention activities that align with the pillars of a Healthy University.

## Methods

### Study Setting

The study setting is QU, a public university located in the urban area of Doha, the capital of the State of Qatar. QU is the primary institution of higher education in the country, serving most of its college-aged population. During the 2021-22 academic year, a total of 24,988 unique students were registered (both full-time and part-time) across 10 colleges, 4 of which are part of the Health Sector (Health Sciences, Pharmacy, Medicine, and Dental Medicine). The majority of these students were at the undergraduate level (foundation or bachelor), comprising 22,699 out of 24,988 (90.84%). Most students were Qatari nationals (17,172/24,988, 68.72%) and female (18,222/24,988, 72.92%) [47].

### Ethics Approval

All studies described below received approval from the QU Institutional Review Board (reference number QU-IRB 1649-EA/22).

### Needs Assessments From Students’ and Key Informants’ Perspectives (Objective 1)

To achieve this objective, we adopted an explanatory sequential mixed methods study design. Specifically, quantitative data will be collected and analyzed first, followed by the gathering of qualitative data to gain an in-depth understanding of QU students’ health status and needs. This design allows for the triangulation of data from both phases, thereby enhancing the validity, reliability, and objectivity of the study results [48]. The findings from the quantitative assessment will help identify priority health issues and their determinants in terms of burden, impact, and unmet needs. The qualitative assessment will then focus on these priority health issues to explore the different contexts and factors shaping them. Additionally, the findings from the quantitative assessment will inform and guide the development of interview guides for the focus groups (FGs) and individual interviews.

The *quantitative assessment* of students’ sociodemographic characteristics, general health status (including mental health), lifestyle (physical activity, diet, and smoking), oral health, health literacy, and health care utilization will be based on data collected through a web-based survey (currently ongoing). The analyses will include both descriptive analyses (eg, the prevalence of risk factors) and associative or comparative analyses (eg, associations between sociodemographic factors and risk factors). In the *qualitative assessment* phase, we will adopt a constructivist interpretative framework, conducting FG discussions and semistructured interviews with students, faculty, and key informants. This approach will help gain a deeper understanding of their perspectives on services and policies that need to be implemented on campus to ensure the physical and mental health and well-being of students at QU [49].

### Quantitative Assessment

A multidisciplinary team of faculty and students from the QU-Health cluster developed the QU Health Survey to conduct an initial assessment of the health status and needs of QU students. The survey consists of 6 sections: demographics, health status (including mental health), lifestyle (physical activity, diet, and smoking), oral health, health literacy, and health care utilization. It was developed using a compilation of validated questionnaires and tools (including some in Arabic), specifically the College Student Health Survey Questionnaire 2021 from Boynton Health, University of Minnesota [50,51]; the International Physical Activity Questionnaire—short form (IPAQ-SF) [52,53]; the 5-item Oral Health Impact Profile (OHIP-5) [54]; the 16-item European Health Literacy Questionnaire (HLS-EU-Q16) [55,56]; and the Youth Health Care measure—satisfaction, utilization, and needs (YHC-SUN) [57]. With the exception of the Boynton Health (college students) and the YHC-SUN (young adults in ambulatory care settings) questionnaires, the IPAQ-SF, OHIP-5, and HLS-EU-Q16 questionnaires were originally developed and validated in representative samples from the general population, rather than specifically among university students. However, all the questionnaires demonstrated adequate levels of reliability (ie, Cronbach α>0.7) during their development and validation phases, and they are widely used across various population segments, including university students [58-60]. Where necessary, the survey questions were translated into Arabic by a research group consisting of QU Public Health faculty members and graduate students. Both the English and Arabic versions underwent pilot testing for clarity and effectiveness with a group of 16 undergraduate students in a public health class. This process led to the rewording of some translated questions.

The health status section includes perceptions of physical and mental health, diagnoses, prescribed medications (including over-the-counter), current counseling received, stress levels, and recent vaccinations. The lifestyle section assesses physical activity (including vigorous- and moderate-intensity exercise, walking, and sitting), use of QU sports facilities, nutrition habits (such as daily consumption frequencies of main food items, breakfast habits, fast-food consumption, and binge eating), and smoking status. The oral health section evaluates self-assessed oral health issues, quality of life related to oral health, and oral health maintenance practices. The health literacy section measures the ease of finding, understanding, and using health information, while the health care utilization section addresses private health insurance, health care service usage, and satisfaction. The survey consists of 70 questions in total, with an estimated completion time of 60 minutes.

### Study Sample Selection and Sample Size Calculation

After obtaining approval from the QU Institutional Review Board, various dissemination methods were used to reach eligible QU students, including advertisements and promotional videos (created by QU-Health students) shared on QU social media, announcements on course Blackboard pages, posters placed across the campus, and mass emails sent to students’ email accounts. This approach resulted in a convenience sample for the study.

Eligibility criteria included students aged 18 years or older with active (part-time or full-time) registration during the Spring and/or Fall semesters of 2022. QU students meeting these criteria formed the target population for the survey (N=24,319).

An initial minimum sample size (*n*) of 385 students was determined to be necessary for estimating a single proportion of 50% (worst-case scenario), using the formula *n*=[*Z*^2^*P*(1–*P*)]/*d*^2^, where Z is the value from the standard normal distribution corresponding to the desired confidence level (ie, 1.96 for a 95% CI), *P* is the expected proportion, and *d* is the desired level of precision (ie, 0.05 or 5%) [61]. To facilitate not only the estimation of single proportions but also the comparison of proportions, means, and/or counts across various outcomes between sociodemographic groups (eg, men vs women, Qatari vs non-Qatari), we aimed to double this sample size. This was accomplished by keeping the dissemination methods active and the web-based survey open for participation throughout the entire calendar year of 2022.

### Data Collection Procedures and Ethical Considerations

The survey was administered as an opt-in web-based questionnaire. Through various dissemination methods, students were provided with a web link to the survey (hosted on the Blue Survey platform; Explorance Inc.). Upon accessing the link, participants were directed to an introductory page outlining the purpose of the survey, information about the researchers and their contact details, eligibility criteria, and the consent form. The consent form emphasized the voluntary nature of the study, participants’ anonymity, the confidentiality of the information collected, and their right to withdraw from the study at any point without consequences. It also highlighted the risks associated with participation. After consenting, participants were granted full access to the survey questions, which could be answered in either Arabic or English. No incentives were provided for participation. To ensure privacy, no identifying information (such as names, emails, or university ID numbers) was collected, and students’ responses could not be tracked. All data were stored in password-protected folders on secure computers, accessible only to authorized researchers.

### Data Analyses

#### Overview

All quantitative data analyses were and will be conducted using Stata version 18 (StataCorp) statistical software.

#### Addressing Selection Bias

**Figure 1:**
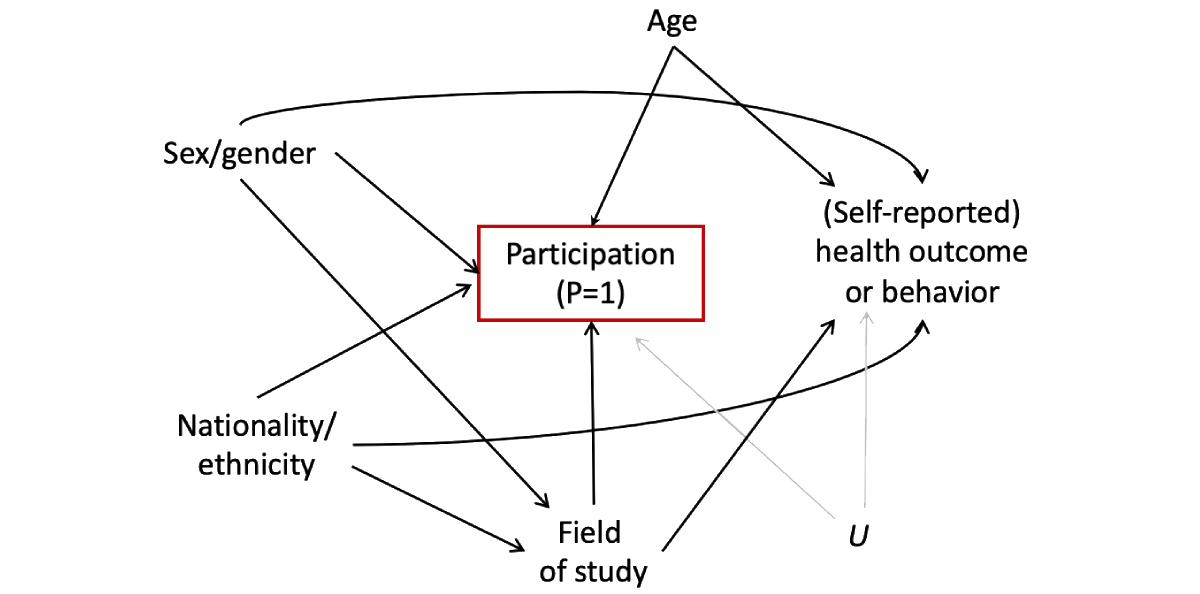
Directed acyclic graph illustrating the threat of selection bias in the present study sample; U, unmeasured or unknown factors.

Data collected from opt-in web-based surveys, such as the one used in this project, are likely to suffer from self-selection bias, as they are limited to those who volunteered to participate [62]. The structure of this bias, a form of collider bias [63,64], is illustrated using a directed acyclic graph (DAG) [65] (Figure 1). We anticipated that factors such as sex/gender, nationality/ethnicity, age, and study field (health-related or not) could influence students’ participation and their self-reported health outcomes or behaviors. Consequently, analyses of data from those who agreed to participate (depicted by the red box in the DAG) will inevitably introduce an artificial association between these characteristics, even if they are unrelated, or bias their correlation with outcomes if they are related in the population under study [66].

To mitigate selection bias and facilitate the generalization of the results from our study sample to the target population [62], we calculated the inverse probability of participation weights (IPPWs) for use in all subsequent analyses [67]. Briefly, the IPPWs were derived from a logistic model that predicted the probability of study participation (*P*=1), conditional on the following variables: gender (1=female vs 0=male), nationality (1=non-Qatari vs 0=Qatari), the field of study (1=health-related vs 0=all other), and academic level (1=postgraduate vs 0=undergraduate), with academic level serving as a proxy for age. All these variables were entered into the model simultaneously, along with all possible 2- and 3-way interactions between them. The inverse of the predicted probabilities, conditional on the predictors, was then used to assign sampling weights to each individual [68].

#### Descriptive and Association Analyses

Study variables will be described as proportions for categorical variables and as means with SDs (or medians with IQRs) for continuous variables, depending on their distribution (normal or skewed). Comparisons of variables based on students’ sociodemographic characteristics (eg, gender, nationality) will be performed using chi-square or Fisher exact tests for categorical variables, *t* tests (unpaired) for normally distributed continuous variables, and Wilcoxon rank-sum tests for nonnormally distributed continuous variables. Depending on the type of outcome under analysis, linear, logistic, or Poisson regression models will be used to examine associations between exposures of interest (eg, sociodemographic characteristics) and study outcomes (eg, health behaviors or status). Results will be expressed as mean differences, odds ratios, risk differences, or relative risks, along with their respective 95% CIs. All analyses will adjust for selection bias using IPPWs, as previously described, and account for potential confounders. Relevant confounders for each association under study will be identified by constructing DAGs using DAGitty Software version 3.0 [69]. A DAG visually represents the assumed causal and often complex relationships between all relevant (measured and unmeasured) variables, helping to identify the minimum sufficient set of variables that must be adjusted for (eg, using multivariable regression methods) to control for confounding the estimates of associations between exposures and outcomes. DAGs are a modern and essential tool in epidemiology that, when comprehensively specified, help identify key confounding variables to consider. This ensures that sufficient adjustments are made to control for confounding, while also avoiding unnecessary (and often harmful) over-adjustments [65].

The reporting of all results will adhere to the STROBE (Strengthening the Reporting of Observational Studies in Epidemiology) guidelines [70].

### Qualitative Assessment

FGs with students and faculty, along with semistructured interviews with other key informants, will be conducted to explore, in greater depth, perceptions of the services and policies that need to be considered on campus to ensure the physical and mental health and well-being of students at QU. This phase will allow the researchers to gain a detailed understanding of the issues that cannot be quantitatively measured within their natural context by engaging directly with students and other key informants [48]. This triangulation of data collection, in terms of both data source (varied participants) and data type (FGs and interviews), allows the phenomenon to be explored from multiple perspectives, thereby enhancing the credibility, dependability, and confirmability of the results [48,49,71].

### Study Sample Selection and Sample Size

For the FGs, purposive sampling will be used to select students from all years of study. Flyers will be posted on campus and shared on QU’s social media pages to recruit participants. To ensure a broad range of perspectives, 2-3 FGs will be conducted with students from the humanities, sciences, business, and engineering colleges, while another 2-3 FGs will be held with students from the QU-Health colleges. Similarly, a purposive sampling of faculty members with more than 3 years of working experience at QU will be recruited. Two FGs will be conducted with faculty members from the humanities, sciences, business, and engineering colleges, while another 2-3 FGs will be held with faculty members from the QU-Health colleges. Each FG will consist of 6-8 participants. The recruitment of students and faculty members will involve selecting participants from various colleges, genders, and nationalities/cultural backgrounds to ensure they reflect the diversity of the QU student and faculty population. FGs will be conducted with students and faculty members until theoretical saturation is reached, meaning no new themes emerge. Based on the literature and previous experience, it is anticipated that 6-8 FG sessions will be necessary to achieve saturation.

### Data Collection Procedures and Ethical Considerations

The FG discussions will center on the perceptions of students and faculty members regarding the health needs and issues that students face, as well as the adaptations required within the university to support their health and well-being. Additionally, the sessions will emphasize the services and policies that should be considered to ensure the physical and mental health and well-being of students, from both the students’ and faculty members’ perspectives. The semistructured interviews will aim to gather in-depth data on the following topics: the major health needs and issues reported by students, the types of services provided to support students’ health and well-being on the QU campus and at the QU Primary Health Care Corporation (PHCC) Center, the policies and regulations implemented to support students’ health and well-being, and the resources and services required to better support students’ health and well-being.

The development of the topic guide will be based on the findings from the qualitative assessment, literature review, and the expertise of the research team, ensuring a systematic approach to data collection. The topic guides will include key open-ended questions, along with probes to allow participants to comfortably express their views on issues related to both the physical and mental health and well-being of students at QU. Once finalized, the topic guides will undergo pilot testing for clarity and effectiveness with a group of students and faculty members from the QU Health.

FGs and interviews will be facilitated by 1 experienced researcher (GFA) who will guide the conversations and discussions within the scope of the topic, ensuring consistency and quality in data collection while minimizing biases. Another researcher (HK) will be present during the data collection sessions to take field notes, as necessary. The FGs and interviews will be conducted in either Arabic or English, depending on the participants’ preferences, and will take place face-to-face on the QU campus or virtually via the Microsoft Teams platform. Data collection sessions will last approximately 45-60 minutes each and will be audio-recorded (with participants’ consent before the session) and transcribed verbatim. Confidentiality and anonymity will be ensured by using alphanumeric codes and storing all research documents on password-protected laptops. No incentives will be provided for participation.

### Data Analyses

The accuracy of the transcripts will be verified by the FG leader and the researchers’ field notes (for the FGs) as well as by the interviewer (for the interviews). To enhance the trustworthiness of the study, the accuracy of the transcripts from both the FGs and the interviews will also be confirmed through consultation with participants. A copy of the transcript will be sent back to participants for member checking, allowing them to confirm whether the transcripts accurately represent their ideas.

Data will be analyzed using a thematic analysis approach, with codes assigned to common quotes and ideas expressed by participants. These codes will be compared and grouped into different categories of themes and subthemes, both inductively and deductively. Two researchers (GFA and BM) will be involved in the analysis process to ensure the credibility and dependability of the results, with any discrepancies resolved through consensus with a third researcher (HK). To perform the thematic data analysis, the computer-assisted coding software NVivo version 14.23.0 (Lumivero) will be used for data retrieval and to critically evaluate participants’ quotes, assigning them to relevant codes and themes.

Measures of quality and trustworthiness in qualitative research, including credibility, dependability, reflexivity, transferability, and confirmability, will be ensured [72,73]. We will apply peer review to all processes, maintain a database of all research data and records, triangulate the data, and implement appropriate data analysis methods. To enhance the quality of reporting in the qualitative phase, we will adhere to the Standards for Reporting Qualitative Research [73].

### Campus Environmental Scan (Objective 2)

The research team will develop checklists and tools for the environmental scan after reviewing the literature to adapt previous assessment tools and draw on relevant student input. They will collaborate with the QU Healthy University Committee members to ensure a comprehensive assessment is developed. QU-Health students (n=40, with 10 students from each college) and research assistants (n=4), supervised by the research team (n=20, faculty members from QU-Health) and QU Healthy University committee members (n=7), will scan the existing health services, programs, initiatives, and activities available on the QU campus and at the QU PHCC Center. They will map how these services align with the health-promoting university indicators. These services and initiatives include, but are not limited to, those promoting healthy lifestyles, health development, mental health, reproductive health, communicable disease prevention, and regular medical check-ups and screening programs for youth.

In addition, the team will review and analyze health policies and regulations available on the QU website. This will involve searching for, reviewing, and analyzing policies, regulations, guidelines, and violation forms related to promoting a healthy lifestyle on campus. Key areas of focus will include the implementation of a 100% tobacco-free policy, the national nutrition policy concerning the types of foods provided in educational facilities and cafeterias, and the availability of qualified and trained physical education instructors. To ensure a comprehensive search and assessment, the team will consult with the QU Healthy University committee members, who have prior experience in data collection from their involvement in submitting the Healthy University WHO award proposal.

### Plan and Implement Evidence-Based Intervention Activities That Align With the Healthy University Pillars (Objective 3)

**Figure 2:**
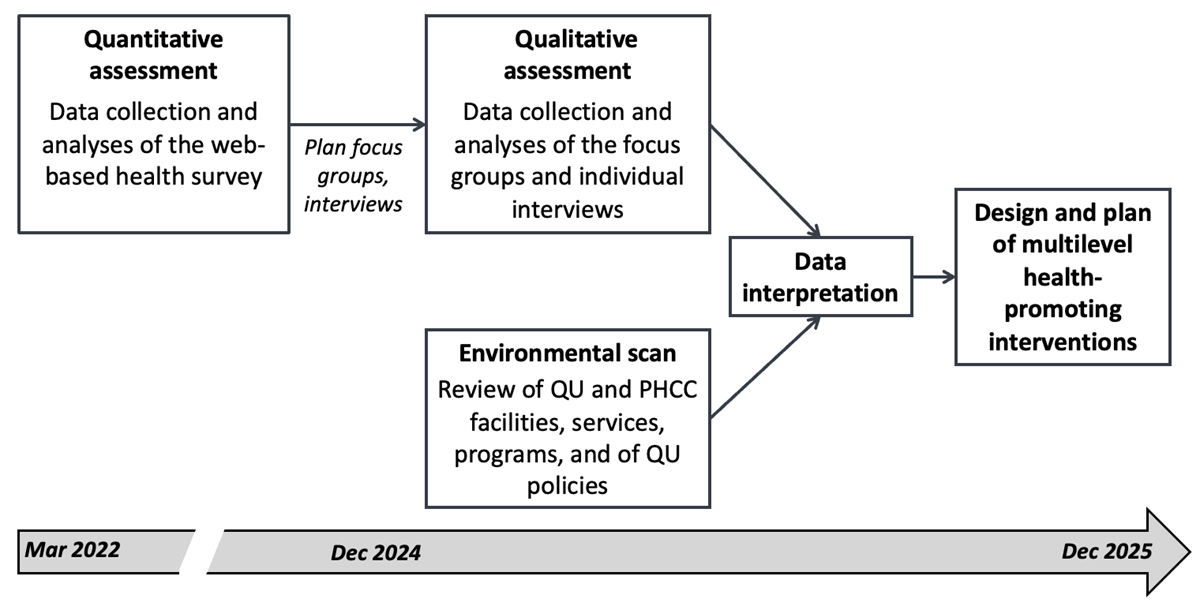
Integration plan of the data from the quantitative and qualitative assessments, and the environmental scan, to inform the design and plan of multilevel health promoting interventions.

Together, the health needs assessments and the environmental scan will provide the evidence necessary to inform and plan effective multilevel interventions and activities tailored to the student’s needs (Figure 2).

The research team members, students, faculty, and both internal and external stakeholders will collaborate to accomplish this objective. Specifically, we plan to achieve the following:

- Organize and deliver health education and promotion activities, initiatives, and programs on campus by organizing campaigns and seminars for students and faculty, both on campus and virtually, with invited international guest speakers from the Health-Promoting Universities network to share their experiences; implementing health and well-being initiatives at the Well-Being Space (a physical space created on campus to support students in practicing hobbies and coping strategies that positively affect their mental health); launching the Peer-helpers program at QU (an initiative between the student learning support and counseling centers to guide students in conducting support groups that empower peers with skills to boost their mental health); and integrating health and well-being modules into core curriculum courses.
- Reorient, where needed, the health services available on campus and at QU PHCC to target the health needs of QU students. This will be achieved by informing and coordinating with all stakeholders (including students, staff, faculty members, the QU PHCC manager, and relevant QU centers’ directors) about the priority health topics identified in the needs assessments. These QU centers include the Career Development Center, the Student Learning Support Center, the Student Counselling Center, the Academic Advising Center, the Special Needs Center, the Facilities and General Services, the Health and Safety Section, and the Strategy and Development Office.
- Reform and/or issue new healthy policies and regulations on campus. This will be achieved by developing health policy recommendations in collaboration with the QU Healthy University Taskforce, which works on collecting data to sustain QU’s Healthy University status.

All findings and initiatives will be shared on QU Health Pulse, a health broadcast service provided by QU-Health to the QU community and beyond, as well as through email and on QU social media platforms.

## Results

Thus far, we have completed the data collection for the quantitative assessment and conducted an initial analysis to assess the extent of potential selection bias. We have also estimated the IPPWs to mitigate this bias in all future data analyses.

**Figure 3:**
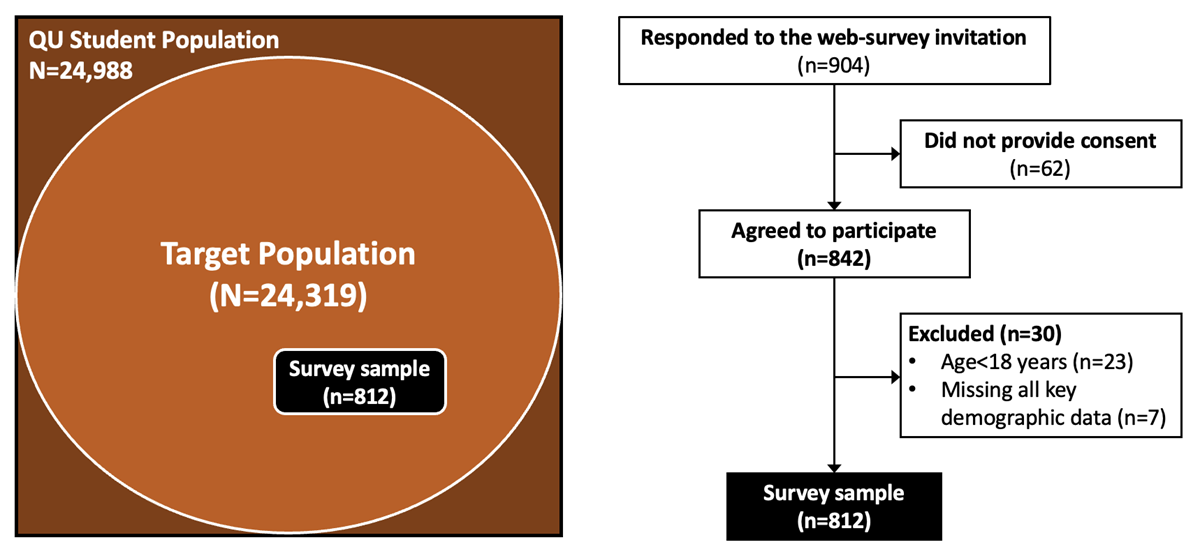
Flowchart describing selection of participants into the quantitative assessment study. QU: Qatar University.

Specifically, during the Spring and Fall semesters of 2022, a total of 904 students responded to the invitation to participate in the QU-health survey, with 842 agreeing to participate. Of these, 30 students were excluded due to failure to meet the eligibility criteria (ie, being younger than 18 years old despite providing consent; n=23) or not providing key demographic data required to generate the IPPWs (ie, age, college, academic level, nationality, and gender; n=7). Therefore, the final study sample for the quantitative assessment consists of 812 students (Figure 3).

Comparisons of the general characteristics of the students included in the study sample and the QU-eligible student population (ie, the target population) revealed evidence of self-selection bias (Table 1). Specifically, students of Qatari nationality were underrepresented (393/812, 48.4%, participants in the study sample vs 16,698/24,319, 68.7%, students in the target population), as were men (125/812, 15.4%, in the study sample vs 6097/24,319, 25.1%, in the target population), particularly Qatari men. Representation across colleges and academic levels was also uneven, with an overrepresentation of students enrolled in QU-Health study fields (232/812, 28.6%, in the study sample vs 1560/24,319, 6.4%, in the target population). However, the academic level of enrollment (undergraduate vs postgraduate), used as a proxy for age, did not differ between the study sample and the target population.

The goal of the IPPW procedure was to make the study sample representative of the eligible population. Indeed, after weighting the data with the IPPWs we recovered the proportions observed in the target population (Table 1), and thus mitigated selection bias in as much as determined by these key variables. These weights will be used in all subsequent quantitative data analyses.

Following the completion of the quantitative data analyses, expected by the end of 2024, the subsequent stages of the project will commence. A detailed study timeline is provided in Table 2.

**Table 1:** Summary statistics of all and the eligible QU^a^ student population, and the study sample, before and after weighting with IPPW^b^.

Variables				All QU students, AY^c^ 2021-22 (N=24,988), n (%)		Eligible QU students (*target population*; N=24,319)^d^, n (%)		Study sample (n=812)		
								Unweighted, n (%)		Weighted (with IPPW), %
**Nationality**										
	Qatari		17,172 (68.72)		16,698 (68.66)		393 (48.40)		68.7	
	Non-Qatari		7816 (31.28)		7621 (31.34)		419 (51.60)		31.3	
**Gender**										
	Male		6277 (25.12)		6097 (25.07)		125 (15.39)		25.1	
	Female		18,711 (74.88)		18,222 (74.93)		687 (84.61)		74.9	
**Gender by nationality**										
	Male Qatari		3599 (14.40)		3484 (14.33)		22 (2.71)		14.3	
	Female Qatari		13,573 (54.32)		13,214 (54.34)		371 (45.69)		54.3	
	Male non-Qatari		2678 (10.72)		2613 (10.74)		103 (12.68)		10.8	
	Female non-Qatari		5138 (20.56)		5008 (20.59)		316 (38.92)		20.6	
**Academic level**										
	Undergraduate		22,699 (90.84)		22,030 (90.59)		739 (91.01)		90.6	
	Postgraduate^e^		2289 (9.16)		2289 (9.41)		73 (8.99)		9.4	
**College (field of study)**										
	**QU-Health^f^**		1602 (6.41)		1560 (6.41)		232 (28.57)		6.4	
		Health sciences	664 (2.66)		641 (2.64)		100 (12.32)			
		Medicine	501 (2.00)		490 (2.01)		60 (7.39)			
		Pharmacy	324 (1.30)		318 (1.31)		52 (6.40)			
		Dental medicine	98 (0.39)		96 (0.39)		20 (2.46)			
	QU-Health^g^		15 (0.06)		15 (0.06)		N/A^h^		N/A	
	**All other**		23,386 (93.59)		22,759 (93.59)		580 (71.43)		93.6	
		Arts and sciences	6914 (27.67)		6720 (27.63)		199 (24.51)			
		Sharia and Islamic studies	1627 (6.51)		1591 (6.54)		62 (7.64)			
		Business and economics	4587 (18.36)		4477 (18.41)		102 (12.56)			
		Education	3156 (12.63)		3073 (12.64)		68 (8.37)			
		Law	1975 (7.90)		1931 (7.94)		23 (2.83)			
		Engineering	4372 (17.50)		4212 (17.32)		124 (15.27)			
		Undefined^i^	755 (3.02)		755 (3.10)		2 (0.25)			

^a^QU: Qatar University.

^b^IPPW: inverse probability of participation weights.

^c^AY: academic year.

^d^Differs from the total QU population by excluding all those aged under 18 years.

^e^Enrolled in certificate, diploma, master, or PhD programs (or equivalent).

^f^Includes health sciences, pharmacy, medicine, and dental medicine.

^g^Registered at the umbrella college level (QU-Health) only.

^h^N/A: not applicable.

^i^These were all enrolled at the certificate level, available only at non–QU-Health colleges.

**Table 2:** Timeline of the tasks in this study.

Task				Start date		End date
Development of study protocol and ethical approval				September 2021		February 2022
**Health needs assessment**						
	**Quantitative (health survey)**					
		Deployment to students	March 2022		December 2022	
		Data extraction and cleaning (database and codebook building)	January 2023		June 2023	
		Data harmonization and calculation of survey weights	September 2023		December 2023	
		Data analyses and reporting	January 2024		December 2024	
		Dissemination (reports, scientific publications, or presentations)	September 2024		December 2024	
	**Qualitative (focus groups and interviews)**					
		Recruitment and conduit	January 2025		May 2025	
		Transcription and data analyses	May 2025		August 2025	
		Dissemination (reports, scientific publications, or presentations)	September 2025		December 2025	
**Campus environmental scan**						
		Review the literature for existing checklists and tools	September 2024		December 2024	
		Physical (built) environment assessment	January 2025		May 2025	
		Review and analyses of Qatar University policies and regulations (on website)	January 2025		May 2025	
		Dissemination (report or presentations)	June 2025		August 2025	
Plan and propose evidence-based interventions based on findings from health needs assessment and campus environmental scan				September 2025		December 2025
Dissemination (writing final report, presentations, and meetings)				November 2025		December 2025

## Discussion

Through this study, we aim to obtain empirical and comprehensive data on the health status and needs of QU students. The individual-level health and needs assessments, combined with assessments at the organizational, community, and policy levels, will provide the evidence needed to design and implement multilevel health promotion interventions tailored to the student’s needs and likely to achieve more sustainable effects. While the project aims to inform relevant policies and services at QU, it is also expected to generate documented experiences and insights that may be applicable to other universities. Specifically, it will offer guidance on aligning institutional efforts with the WHO’s Healthy University indicators by utilizing the Health-Promoting University socioecological framework [16].

This project will address the gap in local knowledge regarding systematic methods to design, implement, and evaluate context-specific interventions aimed at improving the health and well-being of students in the university setting. While several studies in the region have examined university students’ health-related behaviors, along with their determinants and consequences, significant gaps remain that hinder the translation of these findings into impactful interventions. First, students’ health-related behaviors have often been studied in isolation (eg, focusing on physical activity, dietary practices, tobacco use, or mental health [25,29,30,33,34,74,75]) without adopting a holistic approach that acknowledges the interconnected nature of these behaviors and their shared determinants. Second, these health behaviors are frequently analyzed without considering the structural context, including the policies, programs, and services that shape their trajectory and outcomes. Third, the voices of young people are often absent in the prioritization of issues affecting their well-being. This disconnect results in a gap between what policy makers and institutions consider significant and what students perceive as the most substantial contributors to their mental and physical health burdens [76,77].

This project addresses these gaps in several ways. First, it adopts a holistic approach to designing and evaluating multilevel interventions, incorporating the perspectives and preferences of end users. By using mixed methods, the project seeks to gain an in-depth understanding of the multiple layers of influence on health and well-being. In addition to surveys and interviews, the project will assess the environment from social, physical, and policy perspectives, identifying assets and opportunities for creating healthy environments, as well as areas for improvement. The findings from the needs assessment will guide the design of interventions, which will be cocreated and prioritized by university students—the primary end users. The interventions will be assessed for their effectiveness and impact, providing insights into how they can be enhanced to best suit the context and purpose for which they were designed. Thoughtful and tailored dissemination is also crucial to the success of this project. After studying the needs of the QU community and proposing customized interventions and improvements in policy and services, this project is expected to contribute to a broader body of scholarship on youth well-being. Dissemination will occur not only through scientific publications but also by presenting the findings to university leadership and service providers. Finally, a strength of this project is its alignment with national health objectives and initiatives, including Healthy Cities and Healthy Universities.

The main limitation of this study is that it is conducted at a single university in Qatar. However, given QU’s size (the largest in the country), reach, and diversity of the student body, the findings are relevant to universities with similar demographic profiles and can provide a blueprint to be adapted to other contexts. Furthermore, although the quantitative assessment relies on a convenience sample of QU students, efforts have been made to overcome selection bias and ensure the generalizability of the findings to the entire QU student population. However, data obtained through self-reports or narratives may still be subject to recall bias or other measurement biases, such as social desirability in participants’ responses. Additionally, the cross-sectional design of the survey limits the ability to infer causation between some of the assessed sociodemographic determinants and health status outcomes. Repeated assessments (eg, every other year, if feasible) could help mitigate, at least in part, this limitation.

The potential implications of this research include increased awareness of and prioritization of students’ health needs, as well as more active involvement of their voices in the development of policies and services on campus, primary health care services, and other health education and promotion initiatives at QU. Communicating the study findings and proposed interventions to university leadership across various sectors will ensure that our research aligns with the university’s broader strategies for maintaining Healthy University status. By monitoring the long-term impacts of these interventions, the university positions itself as a regional model for effectively integrating student health and wellness into policy frameworks and infrastructural developments. More broadly, the health promotion model established at the university contributes to the national health strategy, of which health promotion is a cornerstone. From addressing the risk factors of prevalent conditions (such as obesity and diabetes) to providing sustainable and culturally appropriate mental health services, this methodological approach could be replicated in community-based initiatives beyond the university. Indeed, this research plan will generate documented experience and insights that may serve as a model for other public institutions aiming to enhance their health promotion efforts.

In conclusion, this mixed methods study aims to identify and prioritize the health needs of QU students and their determinants, and to inform relevant services and policies targeting these needs. By using comprehensive and context-appropriate methods, this project will contribute to QU’s strategic efforts to become a Health-Promoting University.

## Abbreviations

DAG: directed acyclic graph
FG: focus group
HLS-EU-Q16: 16-item European Health Literacy Questionnaire
IPAQ-SF: International Physical Activity Questionnaire—short form
IPPW: inverse probability of participation weight
OHIP-5: 5-item Oral Health Impact Profile
PHCC: Primary Health Care Corporation
QU: Qatar University
STROBE: Strengthening the Reporting of Observational Studies in Epidemiology
WHO: World Health Organization
YHC-SUN: Youth Health Care measure—satisfaction, utilization, and needs

## Data Availability

The data sets generated and analyzed during this project will be available from the corresponding author upon reasonable request, after the completion of the primary analyses.
